# Vegetable and Fruit Intakes of On-Reserve First Nations Schoolchildren Compared to Canadian Averages and Current Recommendations 

**DOI:** 10.3390/ijerph9041379

**Published:** 2012-04-16

**Authors:** Allison Gates, Rhona M. Hanning, Michelle Gates, Kelly Skinner, Ian D. Martin, Leonard J. S. Tsuji

**Affiliations:** 1 School of Public Health and Health Systems, University of Waterloo, 200 University Ave. West, Waterloo, ON N2L 3G1, Canada; Email: rhanning@uwaterloo.ca (R.M.H.); m2gates@uwaterloo.ca (M.G.); kskinner@uwaterloo.ca (K.S.); ljtsuji@uwaterloo.ca (L.J.S.T.); 2 Propel Centre for Population Health Impact, University of Waterloo, 200 University Ave. West, Waterloo, ON N2L 3G1, Canada; 3 Department of Environment and Resource Studies, University of Waterloo, 200 University Ave. West, Waterloo, ON N2L 3G1, Canada; Email: ianmartin@mac.com

**Keywords:** First Nations, diet, nutrition, vegetables, fruit, children

## Abstract

This study investigated, in on-reserve First Nations (FN) youth in Ontario, Canada, the following: (a) the intakes of vegetable and fruit, “other” foods and relevant nutrients as compared to current recommendations and national averages, (b) current prevalence rates of overweight and obesity and (c) the relationship between latitude and dietary intakes. Twenty-four-hour diet recalls were collected via the Waterloo Web-Based Eating Behaviour Questionnaire (WEB-Q) (n = 443). Heights and weights of participants were self reported using measured values and Body Mass Index was categorized using the International Obesity Task Force cutoffs. Food group and nutrient intakes were compared to current standards, Southern Ontario Food Behaviour data and the Canadian Community Health Survey, Cycle 2.2, using descriptive statistics. Mean vegetable and fruit, fibre and folate intakes were less than current recommendations. Girls aged 14–18 years had mean intakes of vitamin A below current recommendations for this sub-group; for all sub-groups, mean intakes of vegetables and fruit were below Canadian averages. All sub-groups also had intakes of all nutrients and food groups investigated that were less than those observed in non-FN youth from Southern Ontario, with the exception of “other” foods in boys 12–18 years. Prevalence rates of overweight and obesity were 31.8% and 19.6%, respectively, exceeding rates in the general population. Dietary intakes did not vary consistently by latitude (n = 248), as revealed by ANOVA. This study provided a unique investigation of the dietary intakes of on-reserve FN youth in Ontario and revealed poor intakes of vegetables and fruit and related nutrients and high intakes of “other” foods. Prevalence rates of overweight and obesity exceed those of the general population.

## 1. Introduction

Over the past 25 years, the prevalence of overweight and obesity has risen steadily in Canada, reaching epidemic proportions [1,2]. Rates of overweight and obesity in Aboriginal populations are becoming increasingly problematic, more so than in the general population [3,4,5,6]. Children are no exception; Aboriginal children face higher rates of overweight and obesity compared to children of other ethnic backgrounds [7,8]. Research in American Indian children reported rates twice that of the national average for overweight and three times for obesity as compared to the general population [8]. The First Nations Regional Longitudinal Health Survey (RHS) reports on the health of First Nations (FN) people living on-reserve in Canada. The most recent RHS (2008/10) indicated that of FN youths age 12–17 years, 29.9% were overweight and 12.8% were obese [9]. These figures were similar to those found in the previous RHS (2002/03) [9]. For Canadian Aboriginal children age 12–17 years living off-reserve, the 2004 prevalence rates of overweight and obesity were 20% and 21%, respectively [10]. In contrast, for Canadian children as a whole (2–17 years), the figures were 18% and 8%, respectively; a markedly lower combined prevalence (26%) as compared to Canadian Aboriginal children (43% and 41% for on- and off-reserve Aboriginal children, respectively) [9,10]. 

The high incidence of obesity and overweight in Aboriginal populations is of particular concern as obesity is the strongest risk factor for type 2 diabetes, a metabolic disorder for which Aboriginal people may be especially susceptible [11,12,13]. Although the etiology of overweight and obesity in Aboriginal children is complex, dietary intake is an immediate, potentially modifiable contributor. Data from the 2004 Canadian Community Health Survey (CCHS) have specifically identified a relationship between low vegetable and fruit intakes in Canadian children and the incidence of overweight and obesity [10]. A review by Pereira and Ludwig also supported an inverse relationship between dietary fibre intake and body weight or body fat, which may be related to a number of plausible mechanisms [14]. Ultimately, higher dietary fibre intakes may help to promote a healthy body weight by increasing satiation and satiety and/or by influencing metabolic fuel partitioning (increasing fat oxidation and reducing fat storage) [14]. Aboriginal people aged 19–50 years in Canada who participated in the CCHS (Cycle 2.2, n = 561) tended to consume vegetables and fruit at intakes lower than suggested and had diets higher in “other” foods [6]. These data, however, exclude Aboriginal people living on-reserve and do not describe the eating habits of Aboriginal children. An investigation of the vegetable and fruit, “other” foods and related nutrient intakes of FN children and adolescents living on-reserve is warranted to address this knowledge gap.

The aims of this study were to (a) describe the vegetable and fruit and relevant nutrient intakes of FN schoolchildren living on-reserve in Ontario, Canada, compared to current dietary standards, the dietary intakes of non-FN youth in southern Ontario (Southern Ontario Food Behaviour data) and average intakes of Canadian children based on CCHS mean intakes, and (b) assess the impact of community (as a surrogate measure of cost of consumables) on dietary intakes. 

As the price of goods in northern Ontario has been reported to be at least two times that in southern Ontario [15], it is expected that the increasing cost of airfreight associated with shipping goods farther north (Table 1) will impact the consumption of vegetables and fruit and “other” foods intakes in the more northern communities, as the goods become more expensive compared to more southern communities.

**Table 1:** A comparison of grocery prices (non-sale items in Canadian dollars) in southern and northern Ontario, Canada, collected in January 2007, except for Peawanuck data which were collected in July 2005.

Community	Location	Fresh 2% milk (2 L)	Red Delicious Apples (per kg)	Pop (12 cans)	White bread (1 loaf)	White potatoes (10 lbs)
**Southern Ontario**						
Kitchener-Waterloo area ^a,b^	43°30’N 80°32’W	3.88	3.14	4.28	2.12	2.67
Georgina Island ^a^	44°22’N 79°17’W	-	-	-	-	-
Christian Island ^a^ (Beausoleil First Nation)	44°50’N 80°12’W	-	-	-	-	-
**Subarctic Ontario**						
Moose Factory Island ^c,d^ (Moose Cree First Nation)	51°16’N 80°32’W	4.69	4.40	6.99	3.19	7.59
Fort Albany First Nation ^c^	52°15’N 81°35’W	7.15	5.79	14.99	4.35	-
Kashechewan First Nation ^c^	52°18’N 81°37’W	7.15	6.75	-	4.45	-
Attawapiskat First Nation ^c^	52°56’N 82°24’W	7.49	7.19	16.99	4.65	12.59
**Arctic Ontario**						
Peawanuck ^e ^ (Weenusk First Nation)	55°15’N 85°12’W	7.79	Not available	21.00	2.90	12.90

^a^ All these communities are within a 1.5 hour driving radius of Toronto, Ontario, the major economic hub of southern Ontario; ^b^ The prices that are given are the average of three major supermarket chains operating in southern Ontario. The Kitchener-Waterloo area data are given as a proxy of prices for Georgina Island and Christian Island, which are not presented, as large grocery stores are not located on these islands; ^c^ Only one major grocery store chain operates in these First Nations, so these prices were collected from the one store in the community; ^d^ Moose Factory Island is located across from the mainland community of Moosonee. Moosonee is the northern terminus of a rail line; thus, the prices in Moose Factory are much cheaper than those communities further north, as these communities are fly-in only in the ice-freeze season, except for an occasional barge; ^e^ At this time, there were three small locally-operated stores, but only one store was open during the community visit. It should be noted that any prices that are presented are the prices if the goods had they been available in the store, which at that time, they were not.

## 2. Materials and Methods

### 2.1. Participants

Data were collected from FN students who attended school in seven FN communities located in Ontario, Canada (Table 1). The more southern Ojibway communities of Christian and Georgina Islands are isolated [16]; however, ferries and/or airboats transport people from the islands to the mainland and operate daily [17]. Major urban centers are easily accessible once on the mainland. The Cree subarctic communities are accessible only by air year-round (daily flights) and connect to Moosonee, Ontario (the northern terminus of rail transportation in Ontario), and by snow-ice road after freeze-up. Peawanuck is the most northern of all of the Cree communities of this study, and at the time of data collection, the community was serviced only twice a week by airplanes. 

Data were collected in partnership with the Chiefs and Councils (elected local FN governing bodies) and/or Education Authorities (similar to Education Boards, but based in FNs), and with approval from the Office of Research Ethics at the University of Waterloo, Ontario, Canada. As passive parental consent is culturally appropriate and typically used in the participating communities, passive consent was used in the present study, after the content was vetted through the Education Authority and/or principals of the schools. An information letter was sent home by the participating schools with each student prior to the study. The parent/guardian information letter for passive consent asked that the parents not wanting to have their child participate in the study to either contact the school representative (named on the letter, with local phone number), community-based health representative (named on the letter, with local phone number) and/or university team member (named on the letter, with local phone number). It should also be emphasized that participating schoolchildren also needed to provide active consent after logging onto the Waterloo Web-Based Eating Behaviour Questionnaire (WEB-Q), the survey instrument used in data collection. Students could either agree to participate by clicking on the checkmark or not participate by clicking on the X. 

Grades 6–8 students had been previously identified as being capable of completing the WEB-Q with minimal supervision [18]; thus, grades 6–8 students were the main cohort studied; other grades were also included at the request of the participating Education Authorities and/or schools (Table 2). The WEB-Q was not scheduled on Mondays—as the WEB-Q records what was eaten on the previous day—a Monday 24 h dietary recall would provide data on Sunday’s intake, which was not the intent of the present study. Thus, the WEB-Q was scheduled during the period Tuesday to Friday. The present study used convenience samples of all students who attended class the day that the WEB-Q was scheduled. To optimize participation rates, the WEB-Q was never scheduled when there were known school trips or activities. If there were students missing on the original day of the WEB-Q, an attempt was made to have the student complete the WEB-Q on subsequent days when possible (*i.e.*, for some communities, there were multiple sampling days to optimize participation rates; Table 3). It should also be noted that all students present on the day of the WEB-Q participated; no student ever opted out.

**Table 2:** Characteristics of the participants in the descriptive comparison of dietary intakes ^a^.

Sample		Sample Characteristics										
Community (collection dates)	Season, Year	n ^b^	Males			Females			Mean age ± SD	Normal weight (%) ^d^	Over-weight (%) ^d^	Obese (%) ^d^
			n (%)	9–13 years	14–18 years	n (%)	9–13 years	14–18 years				
Attawapiskat (22 February)	Winter, 2006	62	28 (45.2)	22	6	34 (54.8)	30	4	12.5 ± 1.0	17 (38.6)	20 (45.5)	7 (15.9)
Attawapiskat (4 March)	Winter, 2010	69	25 (35.2)	25	0	44 (63.8)	36	8	12.2 ± 1.1	19 (30.2)	20 (31.7)	24 (38.1)
Christian Island (19–20 October)	Autumn, 2004	40	23 (57.5)	23	0	17 (42.5)	17	0	11.8 ± 1.0	17 (47.2)	12 (33.3)	7 (19.4)
Fort Albany (10–12 November)	Winter, 2004	63	28 (44.4)	14	14	35 (55.6)	22	13	13.5 ± 2.0	38 (64.4)	15 (25.4)	6 (10.2)
Fort Albany (12–13 December)	Winter, 2007	50	22 (44.0)	12	10	28 (56.0)	21	7	13.0 ± 1.9	n/a ^d^	n/a ^d^	n/a ^d^
Georgina Island (9 December)	Winter, 2003	12	6 (50.0)	6	0	6 (50.0)	6	0	11.5 ± 0.7	7 (87.5)	0 (0)	1 (12.5)
Kashechewan (26–28 May)	Spring, 2009	43	26 (60.5)	18	8	17 (39.5)	15	2	13.1 ± 1.0	16 (40.0)	17 (42.5)	7 (17.5)
Moose Factory (20–23 February)	Winter, 2007	81	44 (54.3)	10	34	37 (45.7)	14	23	14.4 ± 1.5	43 (65.2)	16 (19.8)	7 (10.6)
Peawanuck (7–8 December)	Winter, 2005	10	4 (40.0)	4	0	6 (60.0)	6	0	11.5 ± 0.9	3 (42.9)	2 (28.6)	2 (28.6)
Peawanuck (1–2 June)	Spring, 2010	13	7 (53.8)	6	1	6 (46.2)	5	1	12.8 ± 0.7	3 (23.1)	5 (38.5)	5 (38.5)
Total	-	443	213 (48.1)	140	73	230 (51.9)	172	58	13.0 ± 1.6	163 (48.5)	107 (31.8)	66 (19.6)

^a^ Height and/or weight information was not available for 107 participants as they did not report their height and/or weight in the Web-Based Eating Behaviour Questionnaire. For these participants, Body Mass Index could not be determined. Participants who reported their height and weight had significantly lower intakes of vitamin A (*p* = 0.012) and “other” foods (*p* = 0.023), and higher intakes of fibre (*p* = 0.007); they did not differ by age or sex; ^b ^Refers to the total sample size, including those who did not report their height and/or weight but for whom dietary data were collected. Actual sample size used in the analyses may differ from that reported in Table 3 for several reasons. Participants who had intakes <500 kcal/day and >6,000 kcal/day were excluded from analyses, as these values suggest implausible energy intakes [19,20]. In addition, participants who did not provide age or sex were excluded from the study; ^c ^Totals and percentages include only those who reported their height and weight; ^d^ Height and weight data were not collected during the Fort Albany Winter 2007 data collection.

**Table 3:** Number and percentage of school-aged children who participated in the study by community.

Community (collection year)	# of First Nations students in attendance who completed the WEB-Q (% participating)	# of First Nations students enrolled in the targeted grades who completed the WEB-Q (% participating)
Attawapiskat (2006)	62 of 62 (100%)	Unavailable
Attawapiskat (2010)	69 of 69 (100%)	69 of 96 (72%)
Christian Island (2004)	44 of 44 (100%)	44 of 50 (88%)
Fort Albany (2004)	66 of 66 (100%)	66 of 77 (86%)
Fort Albany (2007)	50 of 50 (100%)	50 of 60 (83%)
Georgina Island (2003)	12 of 12 (100%)	12 of 14 (86%)
Kashechewan (2009)	43 of 43 (100%)	Unavailable
Moose Factory (2007)	81 of 81 (100%)	81 of 84 (96%)
Peawanuck (2005)	11 of 11 (100%)	11 of 13 (85%)
Peawanuck (2010)	13 of 13 (100%)	13 of 15 (87%)

### 2.2. Vegetable and Fruit and Nutrient Intakes

Intakes of vegetables and fruit, “other” foods, vitamin A, vitamin C, fibre and folate were assessed via the 24-hour dietary recall portion of the WEB-Q [18]. The WEB-Q has been used since 2001 to survey over 20,000 Canadian students [18,21,22,23,24,25]. It has been validated in grade six to eight students, including FN students from Fort Albany, Ontario (n = 25, 2004) [22]. The 24-hour dietary recall component, where students are asked to recall all the foods and beverages they consumed the previous day, has good relative validity when compared to dietitian-administered interviews in both non-Aboriginal and Aboriginal students [22]. The survey includes traditional Aboriginal foods typically consumed in the communities under study. It mimics the multiple-pass technique, a five-step method used by dietitians in face-to-face dietary recalls, which includes probing for forgotten foods, specific quantities and any additions (condiments, spreads, *etc*.) [26]. 

### 2.3. Anthropometric Variables and Body Mass Index Classification

Students were measured for height by a trained research assistant, using a tape measure affixed to a wall. After being measured, students were told their height and they would then enter it into the appropriate section of the WEB-Q. In an effort to maintain confidentiality, students weighed themselves using a portable analog weigh scale. Students would then enter their weight into the appropriate section of the WEB-Q. Although diet and anthropometric measurements were self-reported, the privacy and confidentiality of the WEB-Q as compared to dietitian-administered interviews may have decreased potential social desirability bias [27]. 

Body Mass Index was calculated to the nearest 0.1 kg/m^2^ based on the measured heights and weights self-reported in the WEB-Q. Students were classified into three BMI categories (normal, overweight and obese) based on age- and sex-specific International Obesity Taskforce (IOTF) cut-offs [28]. No participants were classified as underweight, as these cutoffs have yet to be validated in youth [28]. Important to note is the fact that although the BMI cutoffs used in this study were the result of data collected from a heterogeneous worldwide population, these cutoffs may not be completely appropriate for FN youth [28]. There exist, however, no specific BMI cutoffs for FN youth at this time. The IOTF cutoffs used may therefore have incorrectly classified some participants as overweight or obese when they were in fact not over-fat. However, the cutoffs used in this study were relatively conservative; World Health Organization (WHO) cutoffs, for example, would have resulted in even higher prevalences of overweight and obesity in the study population compared to the IOTF cutoffs used [29].

### 2.4. Data Analysis

Twenty-four-hour recall data were analyzed using the ESHA nutrient analysis software (Salem, Oregon, version 7.1), in accordance with the 2007 Canadian Nutrient File (CNF) [30]. The 2007 version of Canada’s Food Guide for First Nations, Inuit and Métis (CFG) and CNF serving size specifications were used to analyze food guide servings of vegetables and fruit and “other” foods [30,31]. Canada’s Food Guide for First Nations, Inuit and Métis is a tool published by Health Canada that provides simple guidelines on a healthy diet [31]. It contains four major food groups, including vegetables and fruit (e.g., vegetables, fruit, juice), grain products (e.g., rice, bread, cereals), milk and alternatives (e.g., milk, yogurt, soy beverage), and meat and alternatives (e.g., meat, poultry, fish, legumes) [31]. For the purposes of this study, “other” foods included those that fit the CNF definition of “other” (including saturated and/or trans fats and oils), foods that do not fit into any food group of CFG, high-fat and/or high-sugar foods (including some bakery products), high-salt and/or high-fat snacks, higher and lower calorie beverages not including 100% juice or milk, and alcohol [30,31]. “Other” foods included neither unsaturated fat or oils, nor water and condiments [30]. Dietary Reference Intakes (DRIs) (1999–2003 version) including the Estimated Average Requirement (EAR) for vitamin A, vitamin C, and folate, and the Adequate Intake (AI) for fibre, specific to age and sex, were used for comparison [32]. Food group intakes were compared to 2007 CFG recommendations (excluding the recommendation for green and orange vegetables) [31] and mean intakes reported in the CCHS [33], specific to age and sex. Both food group and nutrient data were compared to comparable data collected in non-FN youth from southern Ontario (The Southern Ontario Food Behaviour study). The Southern Ontario Food Behaviour study used an earlier version of the WEB-Q and collected almost exclusively (96%) weekday food behavior data from 682 non-FN grade 6–8 students from the Peel Region of southern Ontario. From the Region of Peel, the nine participating schools (Morning Star, Darcel, David Leeder, Lisgar, and Queenston from Mississauga; Larspur, Fernforest, and Greenbriar from Brampton; and Credit View from Caledon) were randomly selected to participate by the Region of Peel Health Unit, and represented a range of income levels and ethnic backgrounds. Data were collected after consent (passive or active) was obtained.

Statistical analyses were carried out using Statistical Package for the Social Sciences (SPSS Inc., Chicago, IL, USA, version 17.0). The p-value of statistical significance for all analyses was 0.05. Descriptive statistics were computed for food group and nutrient intakes, compared to current standards, the Southern Ontario Food Behaviour data and CCHS average intakes by age and sex. Differences in intakes between communities matched for season and year were tested via ANOVA. Because nutrient intakes are influenced by total energy intake in that those who consume greater energy from food also eat, on average, more of all specific nutrients, nutrient and food group intakes were adjusted for energy intake using the nutrient density method, to reflect intakes per 1000 kilocalories (kcal) [34]. Given the small sample sizes used in the comparison of dietary intakes between communities, the more robust Brown-Forsythe statistic was used. The magnitude of the difference between communities was calculated for each food group or nutrient variable where a significant difference was detected with the ANOVA. The magnitude of difference was calculated to reflect the number of reference community mean standard deviations (SDs) (where the reference community was the community with the largest sample size of the pair, as this community would give the best estimate of true variance) as follows:

(matched community mean − reference community mean)/SD of the reference community mean.

Where a significant difference in nutrient or food group intakes between the two communities was not detected, the minimum effect size that could be detected was calculated using the mean square error generated by the ANOVA as an estimate of variability, with alpha and beta equal to 0.10. The minimum detectable effect size was based on the minimum number of reference community SDs according to the following equation:

δ = [(t_α_ + t_β_)(√MSE)(√2/n)]/SD_ref_

δ = minimum detectable effect size,

MSE = mean square error,

n = sample size per community, and

SD_ref_ = standard deviation of the reference community.

## 3. Results

### 3.1. Comparison of Dietary Intakes to Current Dietary Standards, the Southern Ontario Food Behaviour Data and CCHS Food Group Data

A total of 443 students from seven FN communities were included in the descriptive analysis of dietary intakes (Table 2). All students in the grades investigated who were present at school on the day of the survey completed the questionnaire (100% of school-attending youth). Participation rates among the entire population of school-aged youth in the communities of interest (including those who did not attend school on the study days) are shown in Table 3. Prevalence rates of overweight and obesity in the sample population were 31.8% and 19.6%, respectively (total combined prevalence of 51.4%). In all age and sex groups, mean intakes of vegetables and fruit, fibre and folate were less than the current recommendations (Table 4). Mean intakes of vitamin A for females aged 14–18 were also less than current recommendations; males aged 14–18 had the lowest vegetable and fruit intake of the sample. In all age and sex groups, mean intakes of all food groups and nutrients examined were also less than the mean intakes of non-FN students in southern Ontario, with the exception of “other” foods in males aged 14–18. Males and females in all age and sex groups had intakes of vegetables and fruit lower than those reported in the CCHS. The average number of servings of “other” foods was greater than the average number of servings of vegetable and fruit in all age and sex groups. 

**Table 4:** Nutrient and food group intakes as compared to current dietary standards and Southern Ontario Food Behaviour data ^a^.

Nutrient or food group	On-reserve First Nations schoolchildren from northern and southern Ontario				Southern Ontario Food Behaviour Data				Recommen-dations
	Sex (n)	Age group (years)	Median intake	Mean intake ± SD	Sex (n)	Age group (years)	Median intake	Mean intake ± SD	
Vegetables and fruit (servings) ^b^	Male (n = 140)	9–13	2.9	3.5 ± 3.2	Male (n = 260)	9–13	4.3	5.4 ± 4.4	6
“Other” foods (servings)			4.5	5.5 ± 4.4			6.2	6.8 ± 4.2	-
Fibre (g) ^c^			8.6	10.9 ± 8.3			14.8	16.0 ± 9.6	31
Folate (μg) ^d^			178.5	215.5 ± 160.8			313.2	347.2 ± 197.6	250
Vitamin A (RAE) ^d^			406.8	494.7 ± 479.9			801.7	1,315.5 ± 1,599.0	443
Vitamin C (mg) ^d^			72.8	112.0 ± 120.7			115.1	164.6 ± 147.0	39
Vegetables and fruit (servings)	Male (n = 73)	14–18	3.3	3.3 ± 2.7	Male (n = 28)	14–18	4.3	5.5 ± 4.4	8
“Other” foods (servings)			7.0	8.4 ± 5.7			6.1	6.8 ± 4.2	-
Fibre (g)			10.4	11.2 ± 7.6			14.9	16.1 ± 9.6	38
Folate (μg)			244.2	293.1 ± 212.6			312.5	348.1 ± 197.9	330
Vitamin A (RAE)			535.0	656.9 ± 492.8			807.5	1,324.2 ± 1,605.8	630
Vitamin C (mg)			81.5	110.2 ± 108.1			117.2	166.1 ± 147.2	63
Vegetables and fruit (servings)	Female (n = 172)	9–13	2.9	3.6 ± 3.1	Female (n = 336)	9–13	4.5	5.1 ± 3.7	6
“Other” foods (servings)			4.4	5.1 ± 3.8			4.9	5.4 ± 3.5	-
Fibre (g)			9.2	10.0 ± 6.9			12.2	13.5 ± 8.0	26
Folate (μg)			168.1	202.6 ± 171.0			249.4	270.6 ± 153.2	250
Vitamin A (RAE)			370.7	489.4 ± 568.2			675.4	1,210.7 ± 1,450.0	420
Vitamin C (mg)			89.8	120.7 ± 136.6			108.4	141.4 ± 108.6	39
Vegetables and fruit (servings)	Female (n = 58)	14–18	2.7	3.6 ± 3.4	Female (n = 47)	14–18	4.7	5.6 ± 4.0	7
“Other” foods (servings)			4.6	5.6 ± 4.0			6.2	6.3 ± 4.3	-
Fibre (g)			7.7	8.3 ± 6.1			15.2	15.2 ± 9.3	26
Folate (μg)			147.6	204.2 ± 175.0			273.6	274.6 ± 168.3	330
Vitamin A (RAE)			364.4	427.5 ± 330.2			679.6	1,185.9 ± 1,667.9	485
Vitamin C (mg)			87.1	128.6 ± 154.7			124.4	148.2 ± 139.6	56

^a^ Table shows unadjusted dietary intakes; ^b^ Canada’s Food Guide for First Nations, Inuit and Métis minimum servings (Note: National averages for age and sex subgroups are 4.53 and 4.40 servings for boys aged 9–13 years and 14–18 years, respectively and 4.87 and 4.67 servings for girls aged 9–13 years and 14–18 years, respectively); ^c^ Adequate Intake (AI), recognizing that the AI is an estimation of adequacy and not a recommendation, *per se*; ^d^ Estimated Average Requirement (EAR).

### 3.2. Comparison of Intakes by Latitude

Variation in food group and nutrient intakes by community was assessed controlling for season [35] and year [36] (n = 248). The following dataset pairs were investigated: Christian Island and Fort Albany (Autumn 2004), Attawapiskat and Peawanuck (Winter 2005/2006), Moose Factory and Fort Albany (Winter 2007), and Kashechewan and Fort Albany (Spring 2009); these data were collected during the same year and season in communities at different latitudes (Table 5). As specific dataset pairs were included in which conditions were comparable, the study population was not identical to those who participated in the descriptive comparison of dietary intakes (*3.1*). It also included only participants who fell into the 9–13 year-old age group.

**Table 5:** Characteristics of participants in the comparison of dietary intakes by community.

Sample		Sample Characteristics			
Community	Season, Year	n	Mean Age ± SD	Males (%)	Females (%)
Christian Island	Autumn, 2004	40	11.8 ± 1.0	23 (57.5)	17 (42.5)
Fort Albany		36	12.0 ± 0.8	14 (38.9)	22 (61.1)
Attawapiskat	Winter, 2005/06	52	12.2 ± 0.7	22 (42.3)	30 (57.7)
Peawanuck		10	11.5 ± 0.9	4 (40.0)	6 (60.0)
Moose Factory	Winter, 2007	24	12.6 ± 0.5	10 (41.7)	14 (58.3)
Fort Albany		33	11.9 ± 0.9	12 (36.4)	21 (63.6)
Kashechewan	Spring, 2009	33	12.7 ± 0.5	18 (54.5)	15 (45.5)
Fort Albany		20	12.4 ± 0.6	5 (25.0)	15 (75.0)

The comparison of dietary intakes by community is shown in Table 6. In the comparison of Fort Albany and Christian Island, mean adjusted intakes of vegetables and fruit (*p* = 0.039) and fibre (*p* = 0.009) were higher in Christian Island. In the comparison of Peawanuck and Attawapiskat, mean adjusted intakes of fibre (*p* = 0.005) and folate (*p* = 0.029) were higher in Peawanuck. Finally, in the comparison of Fort Albany and Kashechewan, mean adjusted intakes of vitamin C (*p* = 0.011) were higher in Fort Albany.

## 4. Discussion

In this sample of FN youth from Ontario, mean intakes of vegetables and fruit, fibre and folate fell below the current recommendations in all age and sex sub-groups. Results also indicated that the mean vegetables and fruit, fibre, folate, vitamin A and vitamin C intakes of this sample of FN youth from Ontario were less than non-FN students from southern Ontario. Mean intakes of vegetables and fruit in all age and sex groups were lower than CCHS averages. In contrast, mean intakes of “other” foods exceeded vegetable and fruit intakes in all age and sex sub-groups. This is concerning as “other” foods are not a major source of micronutrients, being generally high in fat, sugar and sodium, and may displace the intake of more nutritious foods including vegetables and fruit. The dietary patterns of the youth involved in this study, characterized mainly by poor intakes of vegetables and fruit and excessive intakes of “other” foods, are in line with current research involving Canadian and American Aboriginal populations [10,37,38,39].

**Table 6:** Comparison of dietary intakes between communities ^a^.

Food group or nutrient	Community	n	Mean ± SD	*p*-value	Power	Magnitude of difference (# of SDs)	Minimum detectable effect size (# of SDs)
**Autumn 2004**							
Vegetables and fruit (servings)	Christian Island	40	5.1 ± 3.2	**0.039**	0.542	−0.467	-
	Fort Albany	36	4.7 ± 3.5				
“Other” foods (servings)	Christian Island	40	4.4 ± 3.4	0.697	0.067	-	−12.393
	Fort Albany	36	5.0 ± 3.9				
Fibre (g)	Christian Island	40	12.1 ± 8.5	**0.009**	0.745	−0.553	-
	Fort Albany	36	11.2 ± 9.4				
Folate (μg)	Christian Island	40	266.7 ± 167.8	0.081	0.397	-	−206.359
	Fort Albany	36	262.8 ± 151.4				
Vitamin A (RAE)	Christian Island	40	434.8 ± 298.6	0.389	0.144	-	608.543
	Fort Albany	36	595.2 ± 437.4				
Vitamin C (mg)	Christian Island	40	134.9 ± 112.9	0.117	0.344	-	−146.197
	Fort Albany	36	121.0 ± 102.9				
**Winter 2005/2006**							
Vegetables and fruit (servings)	Attawapiskat	52	3.4 ± 3.3	0.834	0.056	-	10.486
	Peawanuck	10	2.7 ± 2.2				
“Other” foods (servings)	Attawapiskat	52	5.7 ± 4.6	0.086	0.309	-	−2.303
	Peawanuck	10	3.3 ± 2.0				
Fibre (g)	Attawapiskat	52	8.1 ± 7.0	**0.005**	0.985	−1.528	-
	Peawanuck	10	10.3 ± 3.9				
Folate (μg)	Attawapiskat	52	278.3 ± 228.6	**0.029**	0.631	0.798	-
	Peawanuck	10	300.0 ± 100.2				
Vitamin A (RAE)	Attawapiskat	52	737.9 ± 949.2	0.086	0.156	-	−689.895
	Peawanuck	10	386.6 ± 215.7				
Vitamin C (mg)	Attawapiskat	52	151.9 ± 196.4	0.964	0.050	-	−2,314.693
	Peawanuck	10	104.9 ± 84.3				
**Winter 2007**							
Vegetables and fruit (servings)	Moose Factory	24	2.5 ± 2.3	0.613	0.079	-	−6.957
	Fort Albany	33	2.3 ± 1.9				
“Other” foods (servings)	Moose Factory	24	7.7 ± 7.7	0.487	0.092	-	−30.132
	Fort Albany	33	4.8 ± 2.9				
Fibre (g)	Moose Factory	24	8.3 ± 6.1	0.836	0.055	-	−40.195
	Fort Albany	33	7.3 ± 5.8				
Folate (μg)	Moose Factory	24	208.2 ± 142.9	0.795	0.058	-	−607.874
	Fort Albany	33	210.3 ± 136.3				
Vitamin A (RAE)	Moose Factory	24	509.8 ± 572.4	0.087	0.382	-	−430.657
	Fort Albany	33	584.1 ± 472.4				
Vitamin C (mg)	Moose Factory	24	101.4 ± 103.1	0.160	0.310	-	84.670
	Fort Albany	33	57.6 ± 72.2				
**Spring 2009**							
Vegetables and fruit (servings)	Kashechewan	33	2.5 ± 2.1	0.082	0.472	-	1.937
	Fort Albany	20	3.7 ± 3.7				
“Other” foods (servings)	Kashechewan	33	5.8 ± 3.2	0.258	0.222	-	−2.807
	Fort Albany	20	3.4 ± 2.8				
Fibre (g)	Kashechewan	33	10.8 ± 5.9	0.146	0.290	-	6.724
	Fort Albany	20	9.9 ± 6.6				
Folate (μg)	Kashechewan	33	286.8 ± 187.0	0.321	0.240	-	260.544
	Fort Albany	20	241.5 ± 201.8				
Vitamin A (RAE)	Kashechewan	33	323.3 ± 298.4	0.226	0.224	-	296.278
	Fort Albany	20	269.3 ± 184.4				
Vitamin C (mg)	Kashechewan	33	80.4 ± 85.0	0.011	0.853	−0.378	44.412
	Fort Albany	20	153.5 ± 120.1				
**Autumn 2004**							
Vegetables and fruit (servings)	Christian Island	40	5.1 ± 3.2	**0.039**	0.542	−0.467	-
	Fort Albany	36	4.7 ± 3.5				
“Other” foods (servings)	Christian Island	40	4.4 ± 3.4	0.697	0.067	-	−12.393
	Fort Albany	36	5.0 ± 3.9				
Fibre (g)	Christian Island	40	12.1 ± 8.5	**0.009**	0.745	−0.553	-
	Fort Albany	36	11.2 ± 9.4				
Folate (μg)	Christian Island	40	266.7 ± 167.8	0.081	0.397	-	−206.359
	Fort Albany	36	262.8 ± 151.4				
Vitamin A (RAE)	Christian Island	40	434.8 ± 298.6	0.389	0.144	-	608.543
	Fort Albany	36	595.2 ± 437.4				
Vitamin C (mg)	Christian Island	40	134.9 ± 112.9	0.117	0.344	-	−146.197
	Fort Albany	36	121.0 ± 102.9				
**Winter 2005/2006**							
Vegetables and fruit (servings)	Attawapiskat	52	3.4 ± 3.3	0.834	0.056	-	10.486
	Peawanuck	10	2.7 ± 2.2				
“Other” foods (servings)	Attawapiskat	52	5.7 ± 4.6	0.086	0.309	-	−2.303
	Peawanuck	10	3.3 ± 2.0				
Fibre (g)	Attawapiskat	52	8.1 ± 7.0	**0.005**	0.985	−1.528	-
	Peawanuck	10	10.3 ± 3.9				
Folate (μg)	Attawapiskat	52	278.3 ± 228.6	**0.029**	0.631	0.798	-
	Peawanuck	10	300.0 ± 100.2				
Vitamin A (RAE)	Attawapiskat	52	737.9 ± 949.2	0.086	0.156	-	−689.895
	Peawanuck	10	386.6 ± 215.7				
Vitamin C (mg)	Attawapiskat	52	151.9 ± 196.4	0.964	0.050	-	−2,314.693
	Peawanuck	10	104.9 ± 84.3				
**Winter 2007**							
Vegetables and fruit (servings)	Moose Factory	24	2.5 ± 2.3	0.613	0.079	-	−6.957
	Fort Albany	33	2.3 ± 1.9				
“Other” foods (servings)	Moose Factory	24	7.7 ± 7.7	0.487	0.092	-	−30.132
	Fort Albany	33	4.8 ± 2.9				
Fibre (g)	Moose Factory	24	8.3 ± 6.1	0.836	0.055	-	−40.195
	Fort Albany	33	7.3 ± 5.8				
Folate (μg)	Moose Factory	24	208.2 ± 142.9	0.795	0.058	-	−607.874
	Fort Albany	33	210.3 ± 136.3				
Vitamin A (RAE)	Moose Factory	24	509.8 ± 572.4	0.087	0.382	-	−430.657
	Fort Albany	33	584.1 ± 472.4				
Vitamin C (mg)	Moose Factory	24	101.4 ± 103.1	0.160	0.310	-	84.670
	Fort Albany	33	57.6 ± 72.2				
**Spring 2009**							
Vegetables and fruit (servings)	Kashechewan	33	2.5 ± 2.1	0.082	0.472	-	1.937
	Fort Albany	20	3.7 ± 3.7				
“Other” foods (servings)	Kashechewan	33	5.8 ± 3.2	0.258	0.222	-	−2.807
	Fort Albany	20	3.4 ± 2.8				
Fibre (g)	Kashechewan	33	10.8 ± 5.9	0.146	0.290	-	6.724
	Fort Albany	20	9.9 ± 6.6				
Folate (μg)	Kashechewan	33	286.8 ± 187.0	0.321	0.240	-	260.544
	Fort Albany	20	241.5 ± 201.8				
Vitamin A (RAE)	Kashechewan	33	323.3 ± 298.4	0.226	0.224	-	296.278
	Fort Albany	20	269.3 ± 184.4				
Vitamin C (mg)	Kashechewan	33	80.4 ± 85.0	**0.011**	0.853	−0.378	44.412
	Fort Albany	20	153.5 ± 120.1				

^a^Table shows unadjusted intakes. Statistically significant p-values are shown in **bold**.

The combined rate of overweight and obesity in the sample (51.4%) is equally concerning, exceeding the figure quoted in the CCHS of 41% for Aboriginal youth aged 12 to 17 years (measured heights and weights; off-reserve data) [10]. According to the CCHS, in the general population, about three out of every 10 youth aged 9 to 17 years are overweight or obese (measured heights and weights) [33]. The combined prevalence of overweight and obesity in the study sample also greatly exceeded this estimate. It is likely that the poor dietary patterns of the children and adolescents participating in this study are only one of the many environmental contributors to excess weight. 

Generally, the nutrition environment in remote, isolated FN communities is poor, with food prices being two to three times those seen in urban centres [15,40]. It is likely that the poor vegetable and fruit intakes seen in the study population are reflective of the numerous environmental barriers to healthy eating in remote and/or isolated FN communities, including food insecurity [41] and inadequate access to reasonably priced, healthy foods of consistent quality [42]. Previous research in Fort Albany, for example, revealed that the high cost, poor quality, and inconsistent availability of fresh produce in the community store were main barriers to healthy eating; preferences for vegetables and fruit and food preparation knowledge were not [42,43]. Often, “other” foods are less expensive and more energy dense, and therefore a top choice for those shopping on a budget [44]; they may also be of more consistent quality because they are more resistant to the harsh conditions of transport. Further research documenting the contributors to the poor nutrition environment in remote and/or isolated FN communities is merited in order to inform policymakers and work toward providing equal access to healthy foods for the residents of these communities. 

The data collected in the present study will help the participating FN communities in designing local strategies to improve the dietary intakes of children and adolescents. One way to help overcome this barrier is through school nutrition programs. The school environment provides the opportunity to target a large proportion of the population at a time when they are still growing and forming nutrition habits [45]. At the time of data collection, Fort Albany FN already had a healthy school snack program in place for numerous years, providing one to one and a half servings of vegetables and fruit per student per day (all Fort Albany datasets). This may have accounted for the relatively high vitamin A and folate intakes in Fort Albany relative to the comparison communities. 

It should be noted that no consistent community variation in vegetable and fruit and “other” foods intakes were noted in the pairs examined in this study. In the comparison of Fort Albany and Christian Island, the higher vegetable and fruit and fibre intakes observed in Christian Island were as expected. Despite the healthy snack program in Fort Albany, such a program cannot negate the higher costs of healthy food, including fresh produce, in this remote, isolated community. As reported in an earlier study by Gates *et al.* (2011), the high cost and lack of availability of healthy food at the store in Fort Albany remain serious impediments to healthy eating in the community despite the school snack program, as reported by parents [42]. The lower cost and greater availability of healthy food in Christian Island is a plausible contributor to the higher intakes of vegetables and fruit and fibre in this population. In the comparison of Attawapiksat and Peawanuck, the higher intakes of fibre and folate observed in Peawanuck were surprising, given that at the time of data collection, Peawanuck only had one small store. However, given that the intakes of vegetables and fruit in the Peawanuck sample were in fact lower than those observed in Attawapiskat, it is likely that these higher intakes were related not to vegetable and fruit intakes, but to differences in intakes of other food sources of these nutrients (e.g., fortified bread and cereal products). Finally, in the comparison of Fort Albany and Kashechewan, the higher intakes of vitamin C observed in Fort Albany were as expected. At the time of data collection, Kashechewan did not have a snack program in place, while Fort Albany, as previously mentioned, has had one in place for many years. The higher intakes of vitamin C in Fort Albany were likely reflective of the higher intakes of vegetables and fruit in this sample, even though this difference not reach statistical significance. These results suggest that dietary intakes may vary significantly by latitude, as would be expected owing to differences in food prices and availability (*i.e.*, more northern latitudes would be expected to have poorer access to healthy foods). The fact that further differences were not observed could be the result of further community-level differences that were beyond the scope of this study, and also due to lack of statistical power given the small sample sizes. It is also possible that single day recalls did not fully capture variations in food intake and availability. Most likely, other community-level factors (e.g., school nutrition programs, degree of isolation) may have a stronger influence on dietary intakes than latitude. Further research into other potential determinants of healthy eating in remote, isolated FN communities, beyond latitude, is warranted.

## 5. Conclusions

This study provided a unique investigation into the vegetable and fruit and “other” foods intakes of on-reserve FN youth in Ontario. Overall, results suggest that the FN children and adolescents living on reserve in Ontario have similar dietary patterns to those reported for other off -reserve and on-reserve FN populations [10,37,38,39]; the diet being characterized low vegetable and fruit consumption and intakes of “other” foods exceeding those of vegetables and fruit in terms of servings. The total prevalence rate or overweight and obesity is in excess of that reported by the CCHS [10]. Dietary intakes of vegetables and fruit and “other” foods do not vary consistently by latitude, suggesting the influence of other community level factors. 

The dietary intakes of the participants in the present study, on a whole, are likely obesogenic. School nutrition programs, specifically multi-component programs integrating both a nutrition curriculum and healthy food provision, may be a viable tool to help overcome the barriers to healthy eating in the participating communities. The results of this study provide valuable information for the participating communities, giving them the opportunity to specifically target current deficits in dietary intakes.
